# Paradoxes in practices of inclusion in physical education

**DOI:** 10.3389/fspor.2022.978612

**Published:** 2022-09-13

**Authors:** Corina van Doodewaard, Annelies Knoppers

**Affiliations:** ^1^Research Centre “Movement, School and Sports”, Faculty Movement and Education, Windesheim University of Applied Sciences, Zwolle, Netherlands; ^2^School of Governance, Utrecht University, Utrecht, Netherlands

**Keywords:** gender, inclusion, ethnicity, ability, teachers, paradoxes

## Abstract

The purpose of this paper was to explore how high school PE teachers create norms for inclusion based on gender as it intersected with ethnicity, ability, and effort.

## Introduction

Nabaskues-Lasheras et al. (2020) define inclusion in the context of physical education (PE) as the construction of rich learning environments where all students, regardless of gender, (dis)ability, social class, and race/ethnicity, can experience agency, success, and joy through bodily movement. It requires: (a) respecting and celebrating individual differences; (b) fair and equitable distribution of the benefits of PE; and (c) viewing each pupil as an individual, instead of seeing a class primarily as a collective group. This is easier said than done. Teachers use and create meanings to make sense of inclusion and to define what they see as adequate and normal performances, bodies, and behaviors (Wright, 2004). These definitions of normality or adequacy are often used to define the boundaries of inclusion (van Doodewaard, 2022). Students whose performance and effort are seen to fall within the parameters of normalcy or adequacy in PE are “included.”

PE is an environment where individual differences and heterogeneity often manifest themself in very visible and measured ways (Corcoran et al., 2019; Medcalf and Mackintosh, 2019). A student, for example, cannot participate in wall climbing, dancing, or a relay-race, just by being a knowledgeable bystander. PE is a place where bodies are explicitly used, displayed, and talked about (Paechter, 2003). The focus on the body is also implicated in the (re)construction of gender subjectivities (Sperka et al., 2019). Gendered meanings given to sport and bodies often infiltrate PE classes (Preece and Bullingham, 2022).

Practices labeled as inclusive are, however, filled with tensions concerning “who” is supposed to be included, how inclusion can be organized in PE, how categories can be created that contribute to inclusion and how students can be placed in them (Magnússon, 2019). Little is known how teachers deal with this ambiguity in their practices and how their subsequent daily actions and assumptions might contribute to fewer or more (un)equal opportunities for all students.

### Inclusion in the context of PE

Gender is (re)produced in PE whenever assumptions are made about the gendered capabilities of students, the suitability of sports based on gender categories, and/or the selection of activities and pedagogies based on the gender category of students. Although they were often unaware of this, the teachers participating in the studies we report on here, discursively constructed several inclusion paradoxes. We examined how these paradoxes are reflected in the intersections of gender with other constructions such as those of ability and ethnicity. Our analysis of these paradoxes may suggest they are solid and stable constructions, but in daily practices these paradoxes are never so clear cut. Although teachers tended to use gender and ethnicity as if they were separate homogenous constructs of identity, the results of the various studies revealed intersections between gender, ethnicity, and ability.

### Intersectionality

Crenshaw (1991) was the first to argue that gender intersects with other social axes that are mutually constitutive such as race/ethnicity. An examination of inter-relationships between different axes of oppression such as gender, ability, and ethnicity provides an important corrective to essentializing identity constructs that homogenize social categories (Anthias, 2013; Gillborn, 2015). In the following we present fragments from the various studies that illustrate intersections of gender, ethnicity, and ability.

### Methods

We engaged in a secondary analysis of five studies that revealed some of the dilemmas PE teachers face in their effort to work toward more inclusive PE settings for all students (van Doodewaard, 2022). The studies focused on different pedagogical settings, tools, and contexts to reveal discourses that guided practices of inclusion of over 100 Dutch PE teachers, 33 women and 68 men. One female and three male teachers self-identified as ethnic minority. The teachers taught pupils aged 12–18 in multi-ethnic schools. Video stimulated interviews were used to elicit how these PE teachers navigated and/or managed student differences in their classes and to explore how these practices added to processes of inclusion, exclusion, privileging, and marginalization.

In this article we critically focus on the data that revealed how these teachers struggled with dominant discourses about gender while trying to be inclusive. Our analysis focused on the paradoxes that emerged in their practices of gendered inclusion. We found that practices of inclusion and exclusion intersected with constructions of ability, ethnicity, and effort.

## “She just didn't have what it took”

Teachers used their constructed levels of ability as a pedagogy to modify their teaching practices or to push pupils to adapt to the standards embedded in the curriculum. These teachers did not seem to realize that gender hierarchies were part of their inclusion paradox. While watching videos of their own lessons, teachers labeled the performance of girls as inadequate more often than that of boys.

A teacher pointed to the video and explained:

*And this girl here, S., is a girl who really wants to [participate] but she is not skilled enough. …once, when we practiced gymnastics: she just couldn't manage to get a satisfactory grade at her level. And she felt really bad about it because she really wanted to perform well. But yeah…she just didn't have what it took…* ^1^

By constructing “adequate” skill levels as a truth and basing grades on those levels, the teacher prioritized teaching toward ability levels without questioning the norm of adequacy and skills. In so doing, the teacher subjected the student to a label of inadequacy. The students were categorized according to their perceived skill levels ranging from 0 to 4. Each level had its own guidelines for assigning grades; these guidelines included perceived effort. These levels and criteria for assigning grades seemed to be fixed. Such practices counter the tenets of inclusive teaching, in which respecting and celebrating differences, and viewing students as individuals are part of the main values (Nabaskues-Lasheras et al., 2020). The quote also shows that part of the criteria this teacher used to include or exclude consisted of a judgment of adequate willingness or effort that was subordinated to ability. This disciplining of girls to fixed levels of ability and to levels of willingness or eagerness to participate, was visible in another fragment in which a teacher described the performance of a girl:

*She really wanted it—everybody in the class had already succeeded—she wanted to, but she didn't dare to do it. [Then I] just pushed her over the edge- and then….and then she dared to try again. Maybe it was a bad thing to push her like that, but I did help her be successful; it made her happy and able to join her friends again*.

The practice of “pushing her over the edge” was constructed as a caring practice to help the girl extend her boundaries of effort by daring to take a risk, and consequently being able to participate in the desired and adequate way (like the rest of the group). In this case, inclusion, i.e., joining the rest of the group, was constructed as the reward for acquiescing to being pushed over the limit. How the girl experienced this practice of “inclusion” is not known. The quote also shows how the teacher situated inclusion in the level of the group—to be able to perform like the others. This fragment suggests that the constructed boundary of being included or excluded was based on a teacher's judgement and did not necessarily imply agency of the student. Willingness and possibility of success were constructed as appropriate tools to push students into inclusion.

Many teachers constructed difference in the achievement of the desired, supposedly gender-neutral, norms of adequacy. A teacher explained:

“*I think it's fine for the boys to do and learn the somersault as a whole. But [when working with] girls: you have to break the skill down into more steps or smaller learning parts and guide them.”*

The construction of girls who “learn in a different way” and who are subjected to more guidance and smaller steps in learning complex activities, can be seen as a practice of othering. Not only does it uncover a boyish gender norm that is connected to a “just try and learn” norm, but it also reveals how these teachers saw boys and girls as two separate groups and as a result, may be teaching boys and girls in different ways. The intersection of discourses on gender, ability and resulting pedagogies suggest that inclusion practices were saturated with normative constructions that are known to add to exclusionary practices in PE (Azzarito, 2009; Fagrell et al., 2012; Hill, 2015; Gerdin, 2017). Constructing girls as the ones who “don't have what it takes” reveals the boundaries of teaching practices in the name of inclusion and obstructs the fair and equitable distribution of PE benefits for all. Such constructions illustrate the paradox of engaging in practices that are constructed as inclusive concerning ability but that simultaneously exclude.

## “I just trust these boys slightly less”

The results also revealed that the constructions of inclusion used by the teachers drew on intersecting discourses of gender and ethnicity/race. First, practices of masculinity and boyish behavior were positioned as opposite of that of girls in phrases like: “*Boys want to show what they can do, and girls want to hide their failures”*. The willingness to take risks and showing effort in demonstrating ability, were often constructed as the norm. This norm suggests that engaging in “boyish” behavior would likely result in inclusion. Such judgements become visible a in practices in the selection of (video) role models. Picking adequate (white) boys, seemed to be used as an instrument to reward and acquire cooperative, willing behavior. One of the teachers explained how they selected role models for the videos:

*Well, I had five guys who were always very cooperative during class, who always, yeah…. helped me to set up equipment and… those were really five boys of whom I thought: ‘yeah, they deserve to [do this]….'*.

Other teachers agreed with this method of selecting—and reaffirmed “*Of course, good effort always pays off”*. They seemed unaware of their production of gender in practices of “good effort” and presented these practices as supposedly gender-neutral.

The intersection of ethnicity/race however, revealed the instability of this gender norm of boyish behavior. When verbally reacting to video fragments of their own classes, the teachers constructed non-Western boys as macho, rambunctious pupils that earned respect and high status for their ability. The boys' efforts to be the best did not always mean their skill performance was acceptable, however. A teacher explained:

*Well, you know these tough macho guys want to be the funniest, the best. They are the ones who are in charge, and it is never their fault if they lose*.

By framing the behavior of “these macho guys” as (too) competitive, and at the same time constructing the boys as bad losers, the teacher implicitly constructed a boundary for inclusion and socially positioned the skilled boys on the edge of this boundary. The teachers constructed such behavior as challenging the status quo of their classroom management. This resulted in teachers using disciplinary practices to subject these boys into compliance. Such disciplining practices are however, at odds with values of inclusive teaching practices as described by Nabaskues-Lasheras et al. (2020), meaning respecting individual differences, providing equal opportunities for the distribution of PE for everybody and recognizing every student as a unique human being. As the next fragment shows, the teachers drew on discourses of ethnicity and race to legitimate such disciplining gender practices:

*It's a strong internal drive these “allochtonen” [non-Western immigrant] boys have, you know? It's about…well, in daily life they often say: “What's in it for me?” And in PE they ask: “How do I get the 10 [highest grade possible]? What grade does this jump give me?”*

By constructing competitiveness as a strong internal (biological, cultural) drive, the teachers objectified and othered these boys, and labeled their participation as non-compliant with the norm. This resulted in restrictive strategies, for instance by separating all boys from the girls. Another teacher explained: “*…these boys are not able to work as independently as the girls are. I just trust them [the boys] slightly less; they joke a lot, and they fool around more”*. The intersection of gender and ethnicity seemed to add to practices of valuing girls' behavior as more desirable and easier to manage.

However, teachers applied technologies of intersections of ethnicity and gender in inclusion practices for girls as well. The teachers othered the behavior of “allochtone” girls as challenging the “neutral” cultural norms. For instance, teachers constructed unwillingness to participate or to communicate as contrary to Dutch cultural practice. Ethnic minority girls were constructed as being unwilling to participate and disinterested in PE. This may make them invisible to teachers: “*Yeh, they [immigrant girls] are easy to ignore. It is easy to forget them but that is wrong”*. A teacher blamed the perceived lack of interest on “their group” culture:

*You see that a lot of immigrant girls hang out with each other. And that they often use the excuse that they have their period or that they are not allowed to participate because of their religion. You often see that they encourage each other in avoiding gym class*.

The numerical dominance of male PE teachers in secondary schools was seen as a reason for the lack of participation of girls with a nonwestern background:

*If a male [teacher] wants to talk with them, they behave like dead birds when sitting next to him in the gym. They don't want to engage in a conversation with men*.

Practices of physical proximity such as manual guidance by the teacher during the learning of a somersault, are part of normalized practices in Dutch PE for Western girls. Some of the teachers held the non-western ethnicity of girls responsible for making it impossible to teach them properly. Teachers drew on frames such as: “*[men] coming too close [to a girl] is culturally unacceptable”*. When ethnicity intersected with gender, gender constructions seemed to be more susceptible to ambiguity (Öhman, 2017) resulting in fewer opportunities for girls from a minority background to learn and enjoy PE. These teachers had not learned to creatively work with such cultural constructs.

Another fragment that revealed the intersectionality of ethnicity and gender, affirms how teachers made their truths of appropriate participation congruent with Dutch standards. A teacher explained:

…*. girls, especially “allochtone” girls—they try to get you involved by playing on your emotions by making up beautiful stories. Then they say: “this and that, I don't have to”. …“I'm not allowed to…”. You mustn't fall for that. In a nice and firm manner, you just say: “Well, listen, these are the rules and if you have a problem with that, bring a note from your parents.” And it's the same with these [allochtone] boys: you have to be firm and consistent in enforcing rules. Yeh, clarify the rules and actually follow the rules. And no discussion, never discussion…*

In this fragment, the boundaries of inclusion become very clear as well: “allochtone” students are forced into inclusion. These rules seem to relate to norms of acceptance, assimilation, and discipline that need to be obeyed, which can be identified as practices of whiteness conflated with Dutchness (Weiner, 2015; van Doodewaard and Knoppers, 2018). Such practices of setting the boundaries for cultural or ethnic inclusion, add to dualistic, essentializing and stereotyping forms of othering immigrants and perpetually stigmatize them as the Other (Bhandari, 2020).

## Discussion

### Inclusion paradoxes in teaching practices

Inclusion studies in education and other domains often frame the notion of practices of inclusion as the answer to ensuring equitable outcomes for all and presume inclusion to be a good and positive concept (Penney et al., 2018; Dobusch, 2021). We highlighted the ambiguities that guided inclusion practices of Dutch PE teachers and suggest that the boundaries of inclusion and exclusion are blurred, fluid, and part of a hidden curriculum. In this sense, inclusion may remain a paradox for students and as such may keep them in disempowered positions, with little agency. Nabaskues-Lasheras et al. (2020) suggest inclusion in the context of PE requires rich learning environments for all students to experience agency, success and joy through bodily movement. The results of this study demonstrated the difficulty teachers had in providing such an environment, in part through their privileging of their constructions of gendered Dutchness.

### The “in between” of intersectionality

A privileging of seeming ethnically neutral gender ignores how gender frames intersect with other social categories, increasing the ambiguity concerning practices of inclusion. Where categories intersected in the current studies, paradoxes arose that forced teachers to adjust their criteria for inclusion, such as using the ability level of the group as a tool to enforce a girl into courageous compliance and reward her with inclusion. Similarly, the efforts of boys to compete, to succeed and to have fun, were presented as a lack of assimilation into Dutchness. The teachers engaged in practices of othering and exclusion, by objectifying such behavior as a characteristic of “those boys” and subsequently categorizing it as too competitive and rambunctious. Such fluidity of the criteria for inclusion/exclusion proved to be powerful instruments used to govern and shape boys and girls toward desirable bodies and behaviors, as defined by the teachers. Teachers implicitly blurred the boundaries of inclusion by moving between essentialist constructions of gender, ability and ethnicity and creating multiple criteria to include or exclude. Such practices illustrate how teachers implicitly shifted along the lines and sections of inclusion—privileging intersections of masculinity and Dutchness as an instrument to “solve” their own difficulty of coping with boundaries of inclusion. By doing so, they enforced the norms that underly their constructions of “inclusion-ability”, which at the same time added to practices of exclusion.

### The beautiful between

Inclusion only makes sense against the background of something or someone else being excluded (Dobusch, 2021). This means teachers are always confronted with dilemmas concerning their inclusion practices. Embracing ambiguity and the unpredictability of teaching rather than using fixed dualistic oppositions and norms may offer teachers opportunities to alter the direction of inclusion paradoxes. Butler (2021) has argued that a livable interdependency is an alternative to inclusion and is the opposite of marginalization. By acknowledging interdependency and every body's need for belongingness and recognition, teachers could alter the debate about inclusion in their classrooms and turn it into practices of transclusion (Biesta, 2019). We call this the domain of the “beautiful between”, which embraces the unpredictability of teaching practices rather than standardizing them (van Doodewaard, 2022). The “beautiful between” offers teachers and students opportunities to re-invent and transform their own PE lessons into habit-able, livable and pleasure-able spaces for all (Standal, 2015).

## Data availability statement

The original contributions presented in the study are included in the article/supplementary materials, further inquiries can be directed to the corresponding author/s.

## Ethics statement

Ethical review and approval was not required for the study on human participants in accordance with the local legislation and institutional requirements. The patients/participants provided their written informed consent to participate in this study.

## Author contributions

CD and AK contributed to conception and design of the article and discussed the data and the preliminary versions of the article. CD organized the data and writing of the article. Both authors contributed to manuscript revision, read, and approved the submitted version.

## Conflict of interest

The authors declare that the research was conducted in the absence of any commercial or financial relationships that could be construed as a potential conflict of interest.

## Publisher's note

All claims expressed in this article are solely those of the authors and do not necessarily represent those of their affiliated organizations, or those of the publisher, the editors and the reviewers. Any product that may be evaluated in this article, or claim that may be made by its manufacturer, is not guaranteed or endorsed by the publisher.

## Footnotes

^1^The fragments in this section present students who were constructed as ethnic neutral students by the teachers, which in these cases meant students were seen Dutch and white.
